# Human substantia nigra neurons encode decision outcome and are modulated by categorization uncertainty in an auditory categorization task

**DOI:** 10.14814/phy2.12422

**Published:** 2015-09-28

**Authors:** Robert A McGovern, Andrew K Chan, Charles B Mikell, John P Sheehy, Vincent P Ferrera, Guy M McKhann

**Affiliations:** 1 Department of Neurological Surgery, New York-Presbyterian Hospital, Columbia University Medical CenterNew York, New York; 2 Department of Neuroscience, Columbia UniversityNew York, New York

**Keywords:** Decision making, dopamine, perceptual categorization, single neuron recording, substantia nigra

## Abstract

The ability to categorize stimuli – predator or prey, friend or foe – is an essential feature of the decision-making process. Underlying that ability is the development of an internally generated category boundary to generate decision outcomes. While classic temporal difference reinforcement models assume midbrain dopaminergic neurons underlie the prediction error required to learn boundary location, these neurons also demonstrate a robust response to nonreward incentive stimuli. More recent models suggest that this may reflect a motivational aspect to performing a task which should be accounted for when modeling dopaminergic neuronal behavior. To clarify the role of substantia nigra dopamine neurons in uncertain perceptual decision making, we investigated their behavior using single neuron extracellular recordings in patients with Parkinson's disease undergoing deep brain stimulation. Subjects underwent a simple auditory categorical decision-making task in which they had to classify a tone as either low- or high-pitched relative to an explicit threshold tone and received feedback but no reward. We demonstrate that the activity of human SN dopaminergic neurons is predictive of perceptual categorical decision outcome and is modulated by uncertainty. Neuronal activity was highest during difficult (uncertain) decisions that resulted in correct responses and lowest during easy decisions that resulted in incorrect responses. This pattern of results is more consistent with a “motivational” role with regards to perceptual categorization and suggests that dopamine neurons are most active when critical information – as represented by uncertainty – is available for learning decision boundaries.

## Introduction

Decision making is frequently thought of as a combination of several processes including signal detection, response selection, outcome, or state evaluation and outcome prediction (Bach and Dolan 2012). The ability to categorize a sensory stimulus is a decision-making process that requires attention to the relevant stimulus dimension, comparison of the stimulus to a category boundary, and selection of an appropriate response. Classically, temporal difference reinforcement learning models of action selection posit that the dopaminergic midbrain system encodes a prediction error which is used by the striatum and higher level cortical regions to learn a reward-based cognitive task (Suri 2002). After an unexpected reward is presented, SN neurons release dopamine in a phasic fashion. When a stimulus is conditioned to predict this reward, the phasic increase in neuronal firing rate shifts to occur reliably after stimulus presentation (Schultz 1986, 1998; Schultz et al. 1997). On the other hand, phasic dopaminergic activity after conditioned stimuli can be seen following a variety of nonreward based, incentive stimuli (Strecker and Jacobs 1985; Horvitz 2000), and modern reinforcement-learning models typically include a specific bonus for motivationally relevant stimuli to account for this (Lesaint et al. 2014).

The ability to categorize sensory stimuli also depends, in part, on the confidence one has in placing the stimulus into a category. The uncertainty surrounding the stimulus, or *categorization uncertainty*, can influence the subsequent behavioral choice. Data from human fMRI studies have linked categorization uncertainty with a fronto-thalamocortical-basal ganglia loop involving the medial frontal cortex, dorsomedial thalamus, anterior insula, and ventral striatum (Grinband et al. 2006; Daniel et al. 2011). Midbrain dopamine (DA) neurons (Fiorillo 2003) and ventral striatum (Preuschoff et al. 2006) have been proposed to encode reward uncertainty, specifically. However, both task-based fMRI and single-neuron, non-human primate recordings have also implicated this system in encoding other aspects of uncertainty including categorization (Aron 2004) and sensory uncertainty (De Lafuente and Romo 2011).

In this study, we chose to examine the activity of human substantia nigra (SN) dopaminergic neurons while subjects performed a simple auditory categorization task in which they received verbal feedback in order to attempt to clarify the role of postsensory stimulus dopaminergic release in the decision-making process. The two major goals of the study were to examine the effect of human midbrain dopaminergic output on decision outcome and uncertainty in a simple perceptual categorization task.

## Materials and Methods

Patients with medically intractable Parkinson's disease (PD) who were considered candidates for surgical therapy were approached for participation in the study. To be considered surgical candidates, patients had to be free of major neurological comorbidities (e.g., Parkinson's-plus syndromes), including dementia. Six consecutive patients (3 males, 3 females) receiving deep brain stimulation surgery for PD targeting the subthalamic nucleus (STN) were enrolled. They had a mean age of 58.0 ± 6.2 years and mean disease duration of 10.8 ± 4.7 years (Table 1). All consent and study procedures were conducted with Columbia University Medical Center Institutional Review Board approval, in accordance with state and federal guidelines. All patients provided their own informed consent.

**Table 1:** Subject demographics and operative characteristics. Unified Parkinson's Disease Rating Scale (UPDRS) scores were derived from a standard set of five items of the motor examination portion of the UPDRS (rest tremor, action tremor, hand rigidity, finger taps, hand grip) performed in the office preoperatively (UPDRS Preoperative), after electrode placement in the operating room (UPDRS OR) and in the office postoperatively with the electrodes turned on (UPDRS Postoperative). Each item has a maximum score of 4 points and thus the highest possible score is 20. Not every item was always able to be tested in the operating room and, in those cases, the corresponding item was excluded from the pre and Postoperative total scores to allow for fair comparison. # of passes refers to the total number of passes required for the placement of electrodes. Coordinates refers to the stereotactic mapping coordinates of the subthalamic nucleus in reference to the intercommissural line connecting anterior and posterior commissures (lateral to intercommissural line, posterior to the midcommissural point, and inferior to intercommisural line, respectively

Patient	Side	Age	UPDRS Preoperative	UPDRS OR	UPDRS Postoperative	# of Passes	Coordinates (mm)
1	Bilateral	52	8	7	4	5	L 9.5, 5, 3
							R 9.5, 5, 2
2	Bilateral	66	9	N/A	6	6	L 12, 4, 2
							R 12, 4, 4
3	Right	54	6	5	1	1	11, 4, 2
4	Bilateral	61	4	1.5	1.5	4	L 11, 5, 2
							R 9, 5, 2
5	Bilateral	52	7	6.5	2.5	2	L 10, 5, 2
							R 12.4, 5, 4
6	Bilateral	62	2.5	2	0.5	2	L 11, 4, 4
							R 11, 4, 2

**Figure 1:**
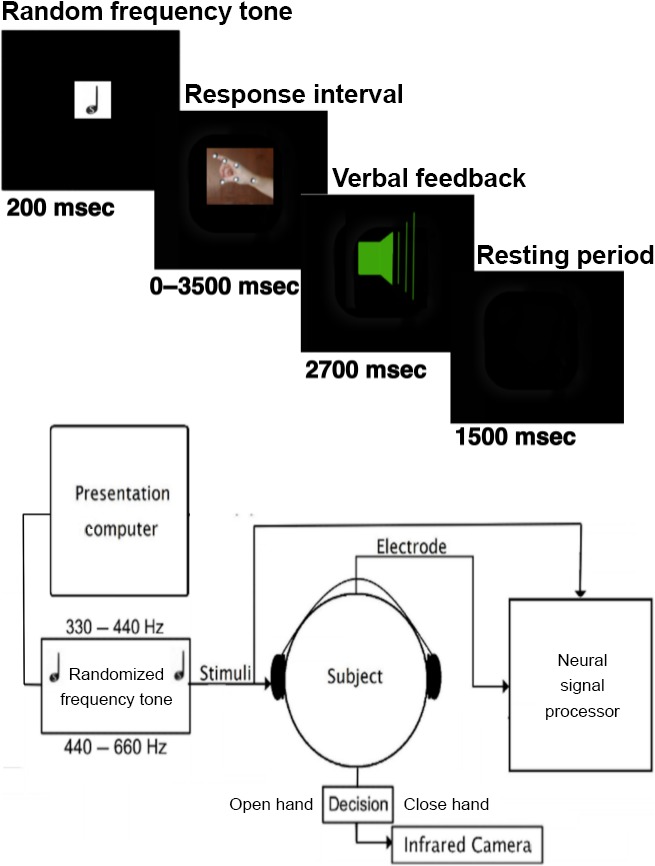
Task schematic and apparatus. (A) Auditory decision-making task. Subjects are instructed to signal whether the tone was high or low compared to an explicitly provided threshold tone with hand opening or closing, respectively. They are then presented with a random tone (Epoch 1, 200 msec) and are given 3.5 sec to respond (Epoch 2). Subjects are subsequently given verbal feedback for 2.7 sec about the correct response (Epoch 3) and have a 1.5 sec rest period before the next tone (Epoch 4). (B) Experimental Apparatus. The apparatus consisted of a presentation computer, headphones, a microelectrode, a neural signal processor, and an infrared camera to record subject response. Although not pictured, the auditory cues and motor responses were also recorded on the same computer for offline analysis.

The STN was identified on preoperative volumetric T1 and T2 MRI using standard neurosurgical techniques. These included direct anatomical visualization of the STN on T2 weighted coronal volumetric images, combined with targeting relative to the midpoint of the plane between the anterior and posterior commissures (Table 1). Microelectrode recordings were carried out with paired 1-*μ*mol/L tungsten-tip electrodes with a power-assisted microdrive. STN and substantia nigra (SN) were then mapped based on characteristic firing patterns. Table 1 shows patient and microelectrode mapping details. A neural signal processor (Cerberus™, Blackrock Microsystems, Salt Lake City, UT) recorded from the microelectrode at 30 kilosamples/sec. The auditory output of the presentation computer was also recorded on one channel of the neural signal processor to allow for accurate comparison of stimulus times and neural activity. Patients' responses (i.e., hand movements) were recorded by an infrared motion capture recording system (ProReflex, Qualisys, Gothenberg, Sweden) recording at 120 samples/sec via three, infrared reflective stickers placed on the subjects' hand contralateral to the microelectrode recording. The auditory output of the presentation computer was also recorded by the computer running the infrared camera so that movement was synchronized with neuronal activity and stimulus onset. This system is more flexible than a simple button press and allows us to precisely define response time, hesitations, and changes in decisions when made (Fig. 1B).

During recording of the SN, we utilized an auditory, decision-making task (Fig. 1A). Subjects were instructed to listen for a randomly generated frequency tone (duration, 200 msec; logarithmic range, 320–660 Hz (440-Hz threshold condition) or 367–825 Hz (550-Hz threshold condition) and to categorize the tone as either higher or lower than an explicitly pre-experimentally provided threshold tone with an opening or closing, respectively, of the hand contralateral to the side of microelectrode recording. Subjects were given up to 3.5 sec to respond after which subjects were verbally informed of the correct response. (Prerecorded audio, interval 2.7 sec: “The correct response was to [open or close] your hand.”). Subjects subsequently were provided a resting period of 1.5 sec to prepare for the subsequent trial. The number of trials was undertaken on a voluntary basis, so as to maximize the number of trial recordings.

**Figure 2:**
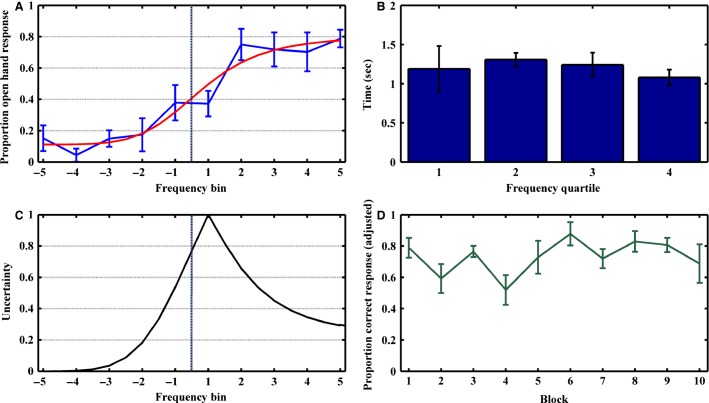
Behavioral results. (A) Proportion of open-hand responses as a function of tone frequency relative to threshold. As the threshold frequency varied for different subjects, frequencies were standardized by sorting them into five logarithmically equally-spaced frequency bins above and below the threshold frequency. Each point represents mean proportion correct for the six subjects for each frequency bin. The vertical black lines represent ± SEM. The black curve represents the psychometric function. The hashed vertical black line represents threshold frequency. (B) To create an uncertainty function (inverted V), the average psychometric function was rectified around the point of subjective equivalence (PSE) and normalized to the range 0 to 1. The PSE is the maximum uncertainty (uncertainty = 1), and the lowest point of uncertainty was set to zero. (C) Response times were greater for near-threshold frequency tone trials, but the effect was not statistically significant. (D) Subject trials were divided into ten equally sized blocks to create a line representing proportion correct response (black line) as a function of cumulative experience across all six subjects. The black vertical lines are ± SEM. Across all subjects, no significant learning occurred throughout the experiment (*F*(9) = 0.67; *R*^2^ = 0.08; *P* = 0.44).

The stimuli consisted of ten to fifteen tones, that were repeated semirandomly so as to ensure an equal presentation of higher- or lower than threshold tones. We used two different threshold frequencies (440 and 550 Hz). Three patients experienced stimuli at one threshold and three patients at the other in order to ensure no perceptual range-dependent effects. The uncertainty value associated with a given tone was derived by creating an uncertainty function (Grinband et al. 2006). The uncertainty function is modeled as a transformation of the psychometric function for the average proportion of open hand response as a function of tone frequency bin (Fig. 2A). The function is rectified around the point of subjective equivalence (PSE) and normalized such that values range from 0 (minimal uncertainty) to 1 (maximal uncertainty). The PSE is set to maximum uncertainty, and the lowest point of uncertainty is set to a value of zero (Fig. 2B). “Difficult” trials were those that included a tone in the two frequency bins immediately flanking threshold (bins −1, 1), and “easy” trials were those included a tone in the two bins furthest from threshold (bins −5, 5).

The recorded microelectrode signals were analyzed offline. A clustering algorithm (Wave Clus, Leicester, U.K.) was used to detect neural spikes and sort them into clusters representing putative neurons (Quiroga et al. 2004). We will hereon refer to clusters as neurons. We defined neurons as dopaminergic in a manner as previously described (Schultz 1986; Mink 1996; Fiorillo et al. 2008). Neurons were considered putatively dopaminergic if their baseline firing rates were between 1.5 and 12 Hz, baseline width (beginning of spike waveform until return to baseline) exceeding 2 msec, peak-to-peak width (distance between two positive waveform peaks) exceeding 0.8 msec, and average waveform consistent with extracellular action potential morphologies from previous mammalian SN neuronal studies (Schultz et al. 1983; Schultz 1986). Patients underwent recording with two electrodes simultaneously and thus multiple neurons were often recorded simultaneously. Activity was measured for each neuron recorded during each stimulus throughout each trial.

We a priori sought to analyze spike activity in the time epoch from 50 to 500 msec after tone onset – an interval that is consistent with response latencies shown in non-human primate single-neuron studies (Schultz et al. 1997; De Lafuente and Romo 2011), and subject response times. To create single-neuron peristimulus time histograms (PSTHs), spike activity was calculated in 1 ms bins, averaged over the total number of trials and then convoluted with a Gaussian kernel (*σ *= 40 msec). We next created neuronal population histograms by calculating the firing rate for each trial, normalizing for baseline activity during the 500 msec prior to tone onset, and averaging over the total number of neurons. To compare across the putatively dopaminergic neurons, the average firing rate was calculated within the 50–500 msec time window for each cell. As the threshold tone frequency was different for different subjects, we standardized frequencies by binning them into five logarithmically equally spaced bins above and below the threshold frequency in order to ensure equal pitch perceptual conditions were represented.

We hypothesized that the neuronal response encodes a linear signal, with higher firing predicting both correct outcome and increasing level of uncertainty. We first analyzed the firing rates of all individual neurons with two-way ANOVA utilizing Matlab software (MATLAB and Statistics Toolbox Release 2011a, The MathWorks, Inc., Natick, MA, 2011 package) in order to classify the neurons as either predictive of outcome, uncertainty, both, or neither (*P* < 0.05). Next, we performed a generalized linear regression analysis on the neuronal population data (MATLAB, glmlab [P. Dunn, 1999]) with the dependent variable being raw firing rate (50-500 ms), and the independent variables being (1) outcome, (2) uncertainty, (3) informed versus uninformed trial, (4) trial number, and (5) motor response (open/closed hand). Outcome, informed versus uninformed trials and motor response were coded as binary variables. Uncertainty and trial number were coded as continuous variables. We utilized an alpha level of 0.05 for statistical significance. We repeated this linear model with nondopaminergic neurons and for the time interval 500–1000 msec after tone onset. Subsequent analyses of variance, chi-squared analyses, simple linear regressions, Wilcoxon rank-sum tests, and descriptive statistics were calculated utilizing Matlab software. Results are reported ± SEM.

## Results

### Behavioral data

Across all recording sessions, the percentage of tones in the 10 frequency bins was uniformly represented (χ^2^(9) = 8.24, *P *=* *0.51). Subjects performed the task with a mean accuracy of 77.5% correct. We examined uncertainty as a function of frequency bin. Trials were sorted into frequency bins to create a line representing proportion open-hand response as a function of tone frequency distance from threshold frequency (Fig. 2A). On average, the maximal uncertainty for the subjects occurred in frequency bins immediately flanking threshold frequency (Fig. 2A, bins 1, −1; proportion correct categorization = 0.37 ± 0.08, 0.62 ± 0.11). Tones with minimal uncertainty occurred in frequency bins furthest from threshold frequency with a left skew (Fig. 2A, bins −4, −5, −3, and 5; proportion correct categorization = 0.96 ± 0.04, 0.85 ± 0.08, and 0.85 ± 0.05, and 0.79 ± 0.06, respectively).

The performance data were fit with a Naka-Rushton function. We plotted the psychometric function for average proportion open-hand response as a function of frequency bin (Fig. 2A) and the uncertainty function by rectifying around the point of subjective equivalence (PSE, bin 1) and normalized such that the range varied from 0 to 1. The PSE is set to maximum uncertainty (bin 1), and the lowest point of uncertainty was set to zero (bin −5) (Fig. 2C). Using this uncertainty function, we were able to assign an uncertainty value to each tone frequency, referred to in subsequent analysis as “uncertainty.”

Average response time over all trials was 1.21 ± 0.08 sec (range, 0.31–2.37 sec). We modeled response time as a function of frequency quartile (Fig. 2B). Frequency quartiles were constructed by bisecting the frequency range above and below threshold tone into two equal bins, respectively. Average response time for the frequency quartiles flanking the threshold frequency, the 2nd and 3rd frequency quartiles, were 1.31 ± 0.09 sec and 1.24 ± 0.15 sec, respectively. These times were nonsignificantly longer than average response times for the 1st and 4th frequency quartiles (1.19 ± 0.29 sec and 1.08 ± 0.10 sec, respectively).

To determine whether behavior was modulated by feedback and experience, we sought to determine the extent of learning during each testing session. Subject mean percent correct response (range, 0.65–0.83, mean, 0.73 ± 0.07) did not change significantly over experimental blocks (Fig. 2D). In addition, there were no significant differences in percent of near-threshold frequencies in a given block (range, 0.42–0.59, mean, 0.53 ± 0.06), ensuring an even distribution of “easy” and “difficult” tone frequencies. Finally, we performed a linear regression adjusting percent correct response by a correction multiplier to account for variability in frequency type per block (correction multiplier = proportion 2nd/3rd quartile frequencies per block/average 2nd/3rd quartile frequencies per block) and the results remained nonsignificant. Thus, our experimental design measures the outcome and uncertainty of an internally constructed stimulus representation without a significant contribution from learning or differences in task difficulty.

Although we found no significant evidence of learning, we sought to determine if outcome or uncertainty was modulated by recent feedback (i.e., the time-order effect). The time-order effect (TOE) posits that exposure to a previous trial that includes a tone in the same category as the current trial, but closer to threshold, is informative because the correct response on the previous trial is the same as the current trial. “Informed” trials were thus defined as those where the tone on the previous trial was between the threshold and the tone on the current trial. If the prior tone was outside the range defined by the threshold and the current tone, the trial was labeled as “uninformed.” In our analysis, we found that subjects had a significantly greater percentage of correct responses on “informed” trials (mean, 89%) relative to “uninformed” trials (mean, 69%) (*χ*^2^(1) = 11.83, *P *<* *0.001).

### Neurophysiological data

**Figure 3:**
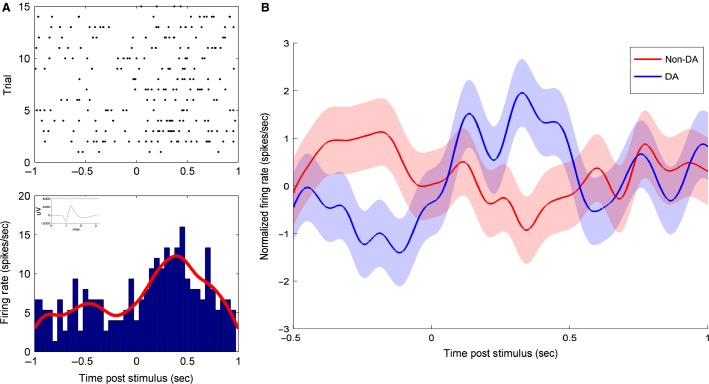
SN dopamine neurons increase their firing rates following cue presentation. (A) Raster plot and peristimulus time histogram (PSTH) for a characteristic SN dopaminergic neuron. The action potential waveform is depicted in the bottom-right inset in blue. This neuron demonstrates a characteristic increase in firing rate 50–500 msec following cue presentation, the a priori period of interest. (B) Mean normalized population histogram firing rate for nondopaminergic and dopaminergic SN neurons. While the population of dopamine neurons demonstrate an increase in firing rate following cue presentation, nondopaminergic neurons show no such increase.

We extracted and sorted single-unit neuronal activity from SN microelectrode recordings yielding 57 spike clusters (3.35 ± 0.37 (SEM) clusters per recording) and retained 34 putatively dopaminergic neurons based on the aforementioned criteria. Figure 3A demonstrates a representative putatively dopaminergic SN neuron with average waveform, PSTH, and its associated raster plot.

We next decided to examine the entire dopaminergic SN neuronal population data. The period from 50 to 500 msec showed significantly elevated raw neuronal firing rate of the entire dopaminergic SN population compared to the baseline period 500 msec prior to tone onset (2.70 ± 0.18 and 2.09 ± 0.14 spikes/sec, respectively; *P *=* *0.009) consistent with a general response to the tone stimulus. After normalizing for baseline firing rate, we compared the population of putatively dopaminergic to nondopaminergic neurons and found that, in contrast to putative dopaminergic neurons, nondopaminergic neuronal firing rate did not appear to increase after tone onset (Fig. 3B).

**Figure 4:**
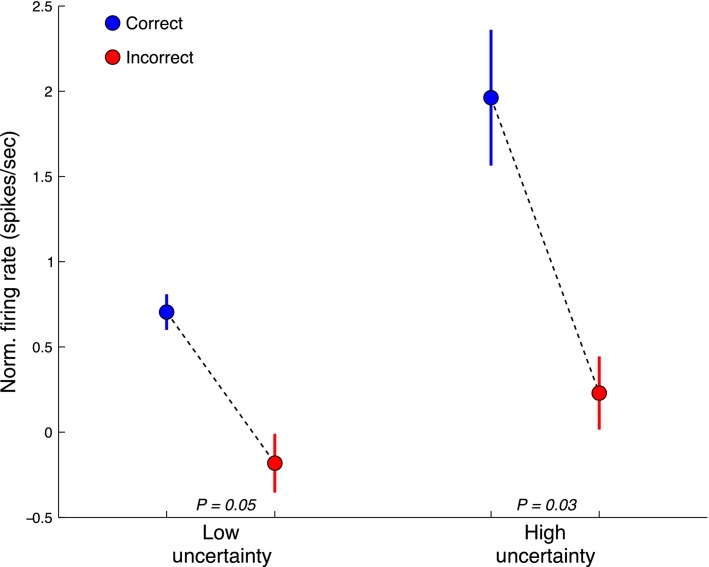
SN dopamine neuronal firing rate correlates with outcome under conditions of both high and low uncertainty. Normalized SN dopaminergic neuronal firing rate is higher for correct decisions under conditions of both high (*P* < 0.03, unpaired *t*-test) and low uncertainty (*P* < 0.05). When accounting for both outcome and uncertainty using a 2-way ANOVA, outcome remained the only significant effect on firing rate (*F* = 8.39, *P* = 0.0039). While the difference in firing rate was increased under highly uncertain conditions (1.73 spikes/sec) relative to low uncertainty (0.89 spikes/sec), this effect (interaction) was not statistically significant although the effect size on outcome was larger when including uncertainty (*F* = 8.39, *P* = 0.0039 vs. *F* = 4.67, *P* = 0.03, respectively).

In order to examine the dopaminergic SN firing rate profile in more detail with respect to our task, we related firing rate to outcome and uncertainty. Figure 4 demonstrates that increased dopaminergic SN neuronal firing rate during the period of interest (50–500 msec postcue) was associated with the correct outcome under conditions of both low and high uncertainty (unpaired *t*-test; *P* = 0.05 low uncertainty, *P* = 0.03 high uncertainty). When examining the effect of uncertainty on firing rate, the effect size appeared to increase, indicating that these cells fired most under conditions of both increased uncertainty while correctly predicting outcome. While firing rate did increase to a greater extent for correct trials under conditions of high uncertainty, this effect (interaction) was not statistically significant although the effect size of outcome did increase with the inclusion of uncertainty (change in firing rate = 1.73 vs. 0.89 (norm spikes/sec); one-way ANOVA for outcome *F* = 4.67, *P* = 0.03; two-way ANOVA including outcome and uncertainty *F* = 8.39, *P* = 0.0039 for outcome only).

**Figure 5:**
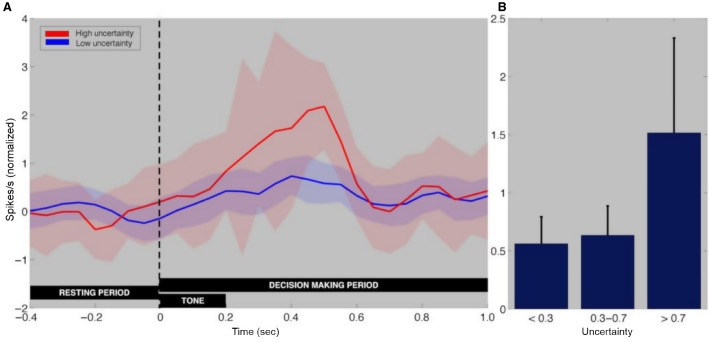
Comparison of SN dopamine neuronal firing rate during uncertain conditions. (A) Mean normalized putative dopaminergic firing rate from −400 msec to 1 sec relative to tone onset for the highest (>0.7; red) and lowest (<0.3; blue) uncertainty trials. The curves depict the mean normalized firing rate across all dopaminergic neurons (*n* = 34) per 50-msec interval by trial type for the period 400 msec to 1 sec relative to tone onset. The shaded area represents ± SEM. (B) Mean normalized firing rate for all dopaminergic neurons for three levels of uncertainty [low (uncertainty < 0.3), medium (0.7 > uncertainty > 0.3), and high (uncertainty >0.7)] in the interval 50–500 msec following tone onset. The bars reflect ± SEM. Average normalized neuronal firing rate reveals a nonsignificant trendwise increase with uncertainty (ANOVA; Low: 0.56 ± 0.23; Medium, 0.63 ± 0.25; High: 1.52 ± 0.82 normalized spikes/sec; *F*(2) Uncertainty = 1.47; *P* = 0.23).

We next compared neural responses to the extremes of uncertainty, that is, firing rates from the frequency bins closest to threshold (-1, 1) to those furthest from threshold (−5, 5). Figure 5A represents the mean normalized SN dopaminergic firing rate from −400 msec to 1 sec relative to tone onset, while Figure 5B demonstrated a nonsignificant trend for higher SN dopaminergic firing rates with increasing uncertainty. Thus, outcome appeared to affect SN dopaminergic firing rate most significantly while uncertainty did not appear to have a statistically significant effect on firing rate. Because neurons in the SN could be subject to other influences, we next proceeded to perform linear regression analyses to account for other potential modulators of firing rate.

We conducted generalized linear model analyses to ascertain the effect of outcome and stimulus uncertainty (our a priori independent predictors of interest) and other potential modulators of neuronal firing rate including trial number in experiment, prior trial experience (informed vs. uninformed), and motor response (open vs. closed hand). We found that, when examining each variable individually, outcome was the only variable that significantly predicted increased dopaminergic neuron firing rate (*P* < 0.03, *n* = 577). When uncertainty was included with outcome, the model explained firing rate significantly better than outcome alone (*P* = 0.0008, *n* = 577). A regression model incorporating outcome, uncertainty, and prior trial effects (i.e., informed vs. uninformed trial type) as predictors of DA neuronal firing rate provided the best fit to the data (*P *<* *0.0001, outcome *β* = 0.66 ± 0.073, uncertainty *β* = 0.79 ± 0.083, informed *β* = −0.154 ± 0.062). We repeated our analysis for the nondopaminergic neurons and for the time interval 500–1000 msec, which were both nonsignificant.

Because we wanted to ensure that this effect was present in individual neurons, we analyzed individual dopaminergic neuronal activity during the period of interest (50–500 msec postcue) by performing two-way ANOVAs with outcome and uncertainty as the main factors. Neurons were then classified into three types based on this analysis: outcome, uncertainty, and outcome–uncertainty. Outcome neurons demonstrated a significant main effect of outcome, but not uncertainty. Uncertainty neurons only demonstrated a significant main effect of uncertainty, while outcome–uncertainty neurons either demonstrated a significant interaction or a significant main effect of both outcome and uncertainty. Overall, 4/34 (12%) neurons were classified as outcome, 6/34 (18%) as uncertainty, and 2/34 (6%) as outcome–uncertainty demonstrating that this effect was not just seen in a model of our dopaminergic SN neuronal population, but in individual neurons as well.

## Discussion

In this auditory decision-making task, subjects had to internally estimate category boundaries to determine the correct response to an auditory tone. The task was designed to examine the choice outcome and uncertainty in categorizing the auditory stimulus in comparison to the internal boundary. Subjects correctly classified tones in 78% of trials with decision uncertainty evident in decreased accuracy as the tone frequency got closer to the defined threshold. A phasic SN dopaminergic neuronal response was seen beginning ˜50 msec after the auditory stimulus and peaking at ˜400 msec. Decision outcome significantly predicted this phasic response while inclusion of uncertainty and prior trial experience significantly improved the fit of a general linear model when added as explanatory variables.

The role of dopamine signaling in decision making has mainly been investigated in terms of reinforcement learning and reward theory. SN neurons release dopamine in a phasic fashion when presented with an unexpected reward. After conditioning, in the absence of reward, SN dopaminergic neurons reduce their firing during the time of expected reward presentation (Schultz et al. 1997; Schultz 1998). This reward prediction error is thought to be the signal encoded by SNPc projections to dorsal striatum in order to learn a cognitive task and underlies temporal difference reinforcement learning models of action selection (Sutton and Barto 1990; Schultz et al. 1997; Guthrie et al. 2009). While the phasic dopaminergic neuronal response has been well described in reward studies, our task allows us to make some observations about this response in the context of a perceptual categorization task. First, this response was seen with a neutral, unpredictable auditory stimulus, confirming prior animal studies examining salient, nonrewarding sensory stimuli (Strecker and Jacobs 1985; Horvitz 2000). In addition, this response is unlikely to be related to novelty as the time and presentation of our auditory stimulus did not vary. Only the tone frequency varied along a relatively restricted auditory spectrum and repetition of stimuli is known to quickly extinguish the novelty response (Ljungberg et al. 1992).

The phasic response was correlated with outcome and occurred prior to action selection, indicating that it was a predictive signal. Non-human primate studies have demonstrated that poststimulus midbrain dopaminergic neuronal firing rate can predict decision choice (Satoh et al. 2003; Morris et al. 2006), activity often described as “chosen value” coding. Prior studies have typically considered decision prediction to be based on reinforcement or machine-learning models of reward-based action selection which use temporal difference errors (reflected in the midbrain dopaminergic firing rate) between predicted states and actual responses to continuously update an algorithm to either directly determine the decision itself (Sutton and Barto 1990; Suri and Schultz 2001) or develop state–response pairs (*Q* values) to predict behavior (Morris et al. 2006). Our study extends this finding by demonstrating this activity during a perceptual categorization task in which no learning was demonstrated throughout the duration of the task.

Why do dopamine neurons continue to produce a phasic response during a task which requires no learning? The most parsimonious explanation is through a motivational or attentional enhancement signal. While reward prediction error can account for the dopaminergic phasic response in reward-based learning tasks, as discussed above, this response is also seen in a variety of tasks related to incentive stimuli and motivation (Redgrave et al. 1999; Horvitz 2000; Robinson and Flagel 2009; Lesaint et al. 2014). Indeed, phasic dopaminergic activity – not reward probability – has been shown to predict choice behavior (Morris et al. 2006). In addition, the phasic dopaminergic response coding for decision outcome is correlated with motivation (as reflected in reaction time) for choices with identical reward expectations in non-human primates (Satoh et al. 2003). Modern reinforcement learning models have incorporated this motivational factor as a bonus for each presenting stimulus (Dayan et al. 2006; Lesaint et al. 2014) to help explain individual differences in the predictive and motivational aspects of conditioned stimuli (Robinson and Flagel 2009). Thus, we propose that this response reflects the intrinsic motivation of subjects to successfully complete the task.

Although we explicitly did not provide a reward such as monetary compensation for correct answers, we did provide feedback after each decision indicating the correct answer for each trial. Thus, positive feedback in this scenario could certainly be construed as a reward, and conversely, negative feedback as punishment. In this manner, our data may be compatible with the conditioning stage of reward prediction theory. During conditioning, phasic dopaminergic output is seen both after the conditioned stimulus and after the presentation of reward (Schultz 1986), similar to the response in our study (Fig. 2B). In the postconditioning stage of reward prediction theory, non-human primates trained to predict reward after a conditioned stimulus do not show an increase in firing rate when that reward is later presented (Schultz 1998). In our study, we continued to see an increase in dopaminergic firing rate when positive feedback was presented (Figure S1). Thus, if we consider the lack of learning in this task to be attributable merely to constraints on time (i.e., subjects would eventually improve with hundreds more trials), then this response may simply be a reflection of the conditioning stage of reward prediction error theory. On the other hand, if subjects' task performance represents a relatively simple task that was quickly learned and conditioned, this possibility is less likely.

While not statistically significant itself, including uncertainty in our generalized linear model improved the model prediction. Functional imaging studies examining various types of decision uncertainty (e.g., reward, categorization) demonstrate a common set of structures activated during uncertain decisions. Midbrain, dorsomedial thalamus, striatum, insula, orbitofrontal, and medial frontal cortex in humans have shown a BOLD response correlated with uncertainty (Grinband et al. 2006; Preuschoff et al. 2006; Abler et al. 2009). Prior non-human primate electrophysiogical studies have demonstrated a slower, sustained tonic dopaminergic output correlated with reward uncertainty (Fiorillo 2003). Interestingly, when examining the neurons only classified as “uncertain” by ANOVA, we saw a similar slower, sustained rise in firing rate during our period of interest when compared to the entire dopaminergic neuronal population (figure not shown). We did not, however, find a statistically significant effect of uncertainty on dopaminergic SN neuronal firing rate overall. Because our study was performed in humans, we were limited in the number of neuronal recordings. Thus, it may be that we were unable to detect a statistically significant tonic dopaminergic signal because there were simply too few neurons from which to record. On the other hand, including uncertainty in our linear model of firing rate clearly improved the fit of the model and increased the effect size of outcome (Fig. 4). Thus, an alternative explanation is that uncertainty increases the overall gain of the outcome signal, enhancing outcome prediction. It is tempting to speculate that this increased signal gain is due to an increase in allocation of attentional resources as subjects need to “pay more attention” to tones closer to the category boundary. In fact, enhanced allocation of attentional resources is a major hypothesis as to the origin of the time order effect (TOE), an important modulator of categorization uncertainty.

The TOE is a well-described phenomenon in stimulus discrimination tasks in which subjects are biased by the order of presentation (Hellstrom 1985; Hairston and Nagarajan 2007). For example, when subjects attempt to categorize a perceptual stimulus, particularly during a two-alternative forced choice task, they tend to be more accurate when the prior stimulus lies closer to the global mean of all presented stimuli than the current stimulus. Subjects are less accurate when the prior stimulus is farther from the global mean than the current stimulus (Karim et al. 2012). This effect suggests that there is greater uncertainty about stimuli that are near the mean, and therefore feedback about responses to these stimuli is more informative. While the neural mechanisms underlying this phenomenon remain poorly understood, some studies have implicated the attentional M3 (or P3 in EEG studies) signal in association with the TOE (Shapiro et al. 2006; Hairston and Nagarajan 2007). Indeed, recent findings by our group have implicated midbrain DA neurons underlying the M3/P3 signal in an auditory oddball task (Mikell et al. 2014). The TOE may thus be a reflection of greater attentional resources being devoted to the more recent stimulus. Subjects in our study did demonstrate the TOE, more accurately classifying tones when the prior trial was “informative.” While including the “information” of the previous trial in our model improved the model fit of firing rate, this seemed to have the weakest effect of all variables included and we cannot conclude that the phasic DA response is related to the TOE.

We observed that an increased SN dopaminergic firing rate correlated with correct decision outcome and was modulated by higher uncertainty despite subjects performing more poorly on more uncertain trials. However, given the likely distinct efferent signals for outcome and uncertainty described above, this result is not unexpected. In fact, SN dopaminergic firing rate was highest during correct, uncertain trials and lowest during incorrect, certain trials. From a functional perspective, this is consistent as trials in which the subject is uncertain but correct likely contains the most relevant information for future trials, whereas trials in which the subject is certain (and therefore the stimulus should be much easier to classify) but incorrect are likely to be errors related to inattention.

There are possible alternative interpretations of this data. Sensory uncertainty could contribute to uncertainty in decision making in this task. However, the tone frequencies were highly discriminable and prior studies have shown that even untrained subjects can detect an approximately 3% difference in auditory frequency (Banai and Ahissar 2004; Bitterman et al. 2008). In addition, in a human fMRI study employing a similar task, using an explicit sensory stimulus as the reference did not change the uncertainty function, suggesting uncertainty was a reflection of internally generated categories (Grinband et al. 2006). We attempted to minimize sensory uncertainty by limiting the frequency of tones to a relatively narrow region. We also changed the threshold tone from 440 to 550 Hz for half of our subjects and saw no difference in subject performance, minimizing the likelihood of sensory uncertainty impacting our results. In addition, though most of the prior literature has examined categorization in terms of learning (Ashby and Ell 2001; Ashby and Maddox 2005; Daniel et al. 2011), we were most interested in determining the role of the midbrain DA system's role in categorical decision making and uncertainty itself rather than the learning process and sought to design a task sufficiently simple that subjects were quickly able to achieve peak performance. We found no evidence that learning affected our results (Fig. 2D). Nevertheless, we cannot exclude a small effect of category learning. Finally, one major limitation of this dataset is that it comes from PD patients. These patients have an underlying degenerative disease process which causes dopaminergic substantia nigra cells to die over time. Thus, the responses seen here may be attenuated compared to healthy subjects or different altogether, given the potentially abnormal underlying neurocircuitry. Nevertheless, these patients represent the only available human subjects who can undergo single neuron recording in the substantia nigra and represent our best efforts at understanding human dopaminergic neurophysiology.

The ability to categorize sensory stimuli represents one of the most fundamental cognitive processes, giving our perceptions meaning. Underlying that ability is the development of an internally generated category boundary and the confidence with which one can place stimuli on either side of that boundary. Our results demonstrate that the activity of human SN dopamine neurons is predictive of perceptual categorical decision outcome and is modulated by uncertainty. Neuronal activity was highest during difficult (uncertain) decisions that resulted in correct responses and lowest during easy decisions that resulted in incorrect responses. This pattern of results suggests that dopamine neurons in the substantia nigra are most active when critical information – as represented by uncertainty – is available for learning decision boundaries.

## Supporting Information

**Figure S1.** SN dopamine neuronal population firing rate following feedback.
