# Supplementary figures and images for: Human substantia nigra neurons encode decision outcome and are modulated by categorization uncertainty in an auditory categorization task
Source: Physiol Rep. 2015 Sep 28;3(9):e12422. doi: 10.14814/phy2.12422 (PMC4600370; doi:10.14814/phy2.12422)

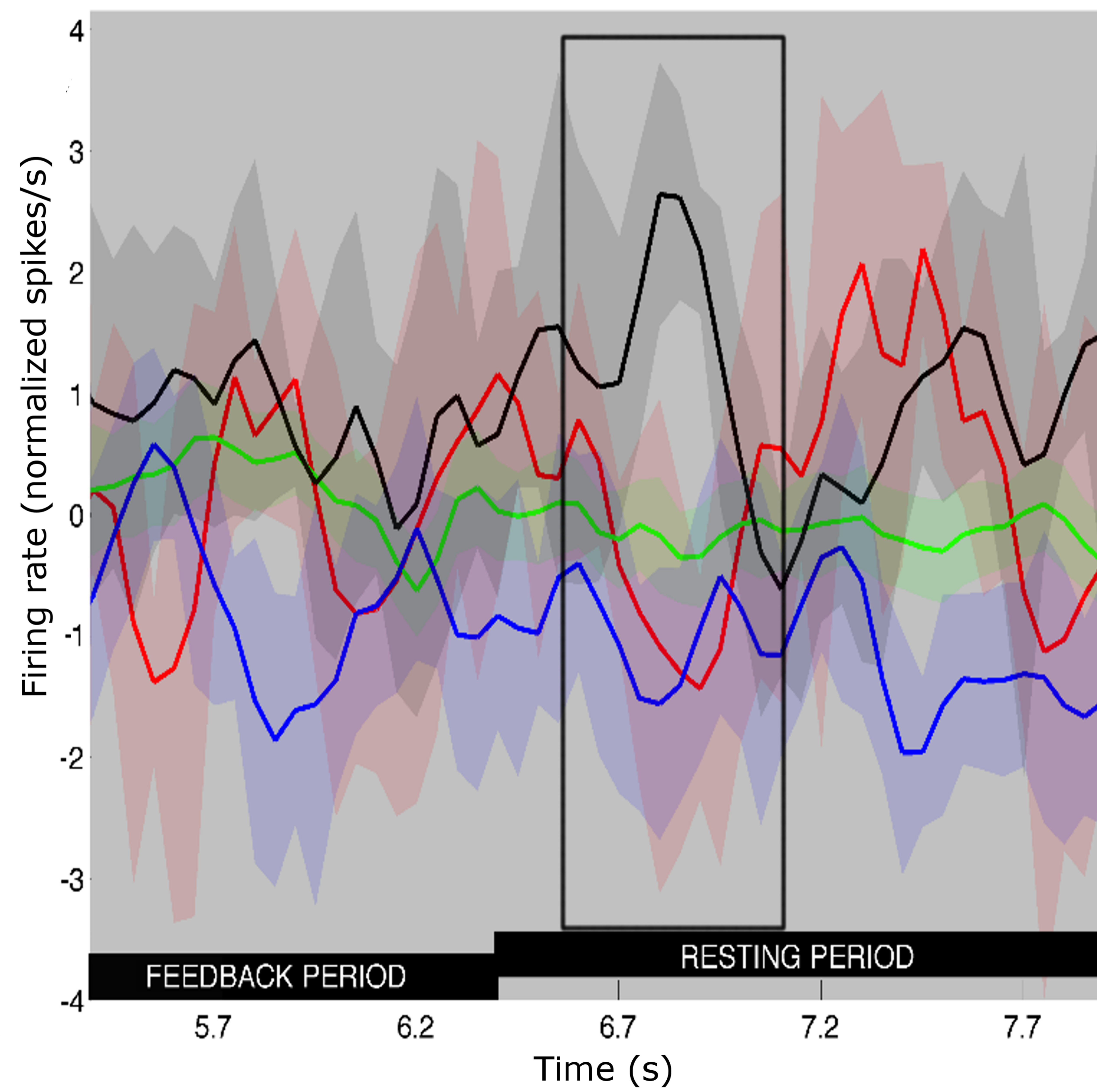

Supplement: Supplementary file 1 [file phy20003-e12422-sd1.pdf]
